# Motivation and incentive preferences of community health officers in Ghana: an economic behavioral experiment approach

**DOI:** 10.1186/s12960-016-0148-1

**Published:** 2016-08-22

**Authors:** Sakiko Shiratori, Enoch Oti Agyekum, Akira Shibanuma, Abraham Oduro, Sumiyo Okawa, Yeetey Enuameh, Junko Yasuoka, Kimiyo Kikuchi, Margaret Gyapong, Seth Owusu-Agyei, Evelyn Ansah, Abraham Hodgson, Masamine Jimba, On behalf of the EMBRACE Implementation Research Team, Yoshiharu Yoneyama, Ebenezer Appiah-Denkyira, Masamine Jimba, Abraham Hodgson, Gloria Quansah Asare, Evelyn Ansah, Junko Yasuoka, Keiko Nanishi, Akira Shibanuma, Kimiyo Kikuchi, Sumiyo Okawa, Margaret Gyapong, Sheila Addei, Vida Kukula, Doris Sarpong, Seth Owusu-Agyei, Kwaku Poku-Asante, Charlotte Tawiah, Yeetey Enuameh, Kwame Adjei, Emmanuel Mahama, Abraham Oduro, Cornelius Debpuur, Francis Yeji, Evelyn Sakeah, Akiko Hagiwara, Sakiko Shiratori, Yusuke Kamiya

**Affiliations:** 1 Japan International Research Center for Agricultural Sciences, 1-1, Ohwashi, Tsukuba, Ibaraki 305-8686 Japan; 2 Japan International Cooperation Agency Health Section, 2nd Floor, The Elizabeth, No. 68A Senchi Link, Airport Residential Area, Accra, Ghana; 3 Department of Community and Global Health, Graduate School of Medicine, The University of Tokyo, 7-3-1 Hongo, Bunkyo-ku, Tokyo, 113-0033 Japan; 4 Navrongo Health Research Centre, P.O. Box 114, Navrongo, Upper-East Region Ghana; 5 Kintampo Health Research Centre, P.O. Box 200, Kintampo, Brong-Ahafo Region Ghana; 6 Research and Development Division, Ghana Health Service, MB 190, Accra, Ghana; 7 Dodowa Health Research Centre, P.O Box DD1, Dodowa, Accra Ghana; 8 Department of Epidemiology & Biostatistics, School of Public Health, Kwame Nkrumah University of Science and Technology, Kumasi, Ghana

**Keywords:** Community health, Discrete choice experiment, Ghana, Health worker, Incentive, Job preference, Mixed logit, Motivation

## Abstract

**Background:**

Health worker shortage in rural areas is one of the biggest problems of the health sector in Ghana and many developing countries. This may be due to fewer incentives and support systems available to attract and retain health workers at the rural level. This study explored the willingness of community health officers (CHOs) to accept and hold rural and community job postings in Ghana.

**Methods:**

A discrete choice experiment was used to estimate the motivation and incentive preferences of CHOs in Ghana. All CHOs working in three Health and Demographic Surveillance System sites in Ghana, 200 in total, were interviewed between December 2012 and January 2013. Respondents were asked to choose from choice sets of job preferences. Four mixed logit models were used for the estimation. The first model considered (a) only the main effect. The other models included interaction terms for (b) gender, (c) number of children under 5 in the household, and (d) years worked at the same community. Moreover, a choice probability simulation was performed.

**Results:**

Mixed logit analyses of the data project a shorter time frame before study leave as the most important motivation for most CHOs (*β* 2.03; 95 % CI 1.69 to 2.36). This is also confirmed by the largest simulated choice probability (29.1 %). The interaction effect of the number of children was significant for education allowance for children (*β* 0.58; 95 % CI 0.24 to 0.93), salary increase (*β* 0.35; 95 % CI 0.03 to 0.67), and housing provision (*β* 0.16; 95 % CI −0.02 to 0.60). Male CHOs had a high affinity for early opportunity to go on study leave (*β* 0.78; 95 % CI −0.06 to 1.62). CHOs who had worked at the same place for a long time greatly valued salary increase (*β* 0.28; 95 % CI 0.09 to 0.47).

**Conclusions:**

To reduce health worker shortage in rural settings, policymakers could provide “needs-specific” motivational packages. They should include career development opportunities such as shorter period of work before study leave and financial policy in the form of salary increase to recruit and retain them.

## Background

Africa’s insufficient health workforce is a major constraint in achieving the health-related Millennium Development Goals (MDGs) [1]. The World Health Organization (WHO) (2006) reported that health worker retention is an important factor in the delivery and quality of health services [2]. The depletion of human resources is particularly serious at the community level in most African countries, including Ghana [3–5].

WHO’s (2010) global policy recommendations on rural retention suggest that countries pursue interventions on four categories: education, regulation, financial incentives, and personal and professional mechanisms [6]. Considering that a range of factors could influence health workers’ motivation, an appropriately selected combination of incentives would be needed to effectively attract and retain health workers to rural areas [7].

Determining health workers’ preferences is an important step to select appropriate incentives [7]. A discrete choice experiment (DCE) has recently been considered a viable strategy to solicit health workers’ preferences for certain job features and practices [8, 9]. A DCE can be used as an attribute-based measure of benefits to describe healthcare interventions, services, or policies according their attributes [10, 11]. This attribute-based measure of benefits helps policymakers develop a national strategy to tackle health worker shortage in rural areas [7, 12].

In Ghana, community health officers (CHOs) who work at community-based health planning and service (CHPS) areas play a crucial role in providing basic health services for community residents. CHOs are trained nurses deployed to communities based on the CHPS Initiative.^1^ Their job covers a wide range of services, including basic preventive care, curative care, and promotional health services in homes or community clinics, and they refer complicated cases to the next level of care [13, 14].

About a half of Ghanaians live in rural areas according to the 2010 Ghana Population and Housing Census [15]. Rural areas in Ghana have limited access to healthcare facilities. Furthermore, poor road infrastructure hinders rural inhabitants’ access to health care in urban centers [16]. Therefore, health services need to be delivered within their communities. It is essential to identify the determinants of CHOs’ willingness to work in order to contribute to the promotion of effective health services. However, shortage, attrition, and low motivation of health workers have been recognized as a challenge for the country [17].

This study explored the motivations and incentive preferences of CHOs through a DCE to promote CHO attraction and retention and, in turn, to improve access to effective community-based health services in Ghana.

## Methods

### Survey design

This study is part of a formative research undertaken prior to the design of an intervention study for the Ensure Mothers and Babies Regular Access to Care (EMBRACE) Implementation Research [18].^2^ EMBRACE is a Maternal Newborn and Child Health (MNCH) initiative with the aim of increasing the uptake of maternal health care as well as reducing maternal and neonatal morbidity and mortality, employing the concept of “continuum of care (CoC)” as a key element. The implementation study was conducted in three Health and Demographic Surveillance System (HDSS) sites: Dodowa, Kintampo, and Navrongo. These sites are respectively located within the Greater Accra (southern), Brong-Ahafo (middle), and Upper-East (northern) belts of Ghana. Most communities (about 73 %) in these HDSS sites are described as rural, and 27 % of them are considered non-rural. According to the 2014 annual report of the Ghana Health Service (GHS), there are about 7210 CHOs in Ghana. Out of which, 1369 (19 %), 560 (7.8), and 484 (6.7 %) had been allocated to the Greater Accra, Brong-Ahafo, and Upper-East regions, respectively [19]. Meanwhile, according to the MOH (2007), about 14,291 CHO were targeted to be deployed into the Ghana Health System by October 2011; thus, considering population growth rate of 2.4 %, 8134 potential CHOs could have been deployed as at 2014 [20].

All the CHOs working in these three HDSS sites were contacted and subsequently recruited for face-to-face interviews and a DCE. The face-to-face interviews used a structured questionnaire that aimed to gather information on the CHOs’ demographic background, professional experience, health service, working conditions, and work attitude. The DCE used a self-administered questionnaire that measured the CHOs’ preference for different job packages.

The survey instruments were jointly designed by the Japanese research team and Ghanaian research team including Ghana Health Service (GHS) and its three Health Research Centers (HRCs). Series of meetings were held involving all partners to develop the survey instruments. We followed the experimental design process’s steps summarized in the literature [21] to ensure the validity. First, we refined the problem. Then we identified the influential attributes as well as their levels to be included through literature review and discussion. Experimental design was generated along with the attributes and their levels considering statistical efficiency. A pretest was conducted in CHPS compounds outside the study area in November 2012. Sixteen CHOs were involved in the pretest: three in Navrongo, five in Kintampo, and eight in Dodowa. Some minor inconsistencies detected during training and pretesting were corrected, and the instruments were finalized before starting data collection.

Data were collected between December 2012 and January 2013. The interviews and experiment were conducted successively. The instruments were not translated into the local languages because all CHOs could speak and understand English. A field supervisor, a research assistant, and a filing clerk checked the data collected by field workers before data entry. The data were then double-entered into EpiData and transferred into STATA ver. 12.1 for processing and analysis. The data from all three sites were merged into one dataset and cleaned.

### Discrete choice experiments

The DCE has become a commonly used instrument in health economics [22]. It is a useful tool to investigate the relative importance to the health worker of different attributes of employment options and to predict their hypothetical choice [23].

In a DCE, respondents are asked to choose between two or more alternatives. Table 1 illustrates an example of the job preference question that the DCE respondents are asked. The DCE determines which incentives would motivate health workers by analyzing their job preference based on the attributes presented in each hypothetical scenario. The DCE results can also be used to calculate the probability that health workers will take a job given certain conditions [7].

**Table 1:** Example of choice set for discrete choice experiment

	Job A	Job B
Salary	Base salary plus 50 %	Base salary
Children’s education	No allowance for children’s education	Allowance for children’s education
Infrastructure, equipment, supplies	Advanced (e.g., reliable electricity, ultrasound, constant drug supply)	Basic (e.g., unreliable electricity, X-ray, intermittent drug supply)
Management style	Unsupportive workplace and management	Supportive workplace and management
Years of work before study leave	Study leave after 2 years of service	Study leave after 5 years of service
Housing	Housing not provided	Free housing provided
Transportation	Utility car provided	Utility car not provided
	☐	☐

Question: Which of these two job postings do you prefer? Select one by checking the box under the job posting you prefer for each choice set

### Theoretical background

A choice experiment is a combination of the characteristics theory of demand [24] and random utility theory [25], implemented through experimental design theory and econometric analysis [22, 26, 27]. The characteristics theory of demand assumes that goods or services can be valued in terms of their constituent characteristics. The random utility model allows us to analyze choice data obtained from respondents’ stated preferences using econometric methods as follows.

The preference of an individual is not embedded in just one factor, but in a combination of factors that may not be readily observable [28]. A utility level *U*_*ij*_ is assigned to each alternative *j = 1*,…, *J* for each CHO *i = 1*,…, *I*. CHOs are assumed to choose the alternative that provides them the highest utility. The utilities are determined by the attributes of both the individuals making the choice and the alternatives available. Not all of those determinants are observed, yet one can separate overall utility into two independent additive parts: a deterministic part (systematic component), *V*_*ij*_, and a stochastic part (random component), *ε*_*ij*_. Then the CHO’s utility becomes

$$ {U}_{ij}={V}_{ij}+{\varepsilon}_{ij} $$

It is then assumed that CHO *i* will choose alternative *j* if and only if that alternative maximizes his or her utility among all *J* alternatives included in the choice set C_*j*_. In this study’s discrete choice experiment, *J* = 2. The probability *P*_*i1*_ that CHO *i* chooses alternative 1 is equal to the probability that the utility *U*_*i1*_ is larger than *U*_*i2*_. The probability that CHO *i* chooses alternative 1 is

$$ \begin{array}{ll}{P}_{i1}\hfill & =\mathrm{Prob}\left({U}_{i1}>{U}_{i2}\right)\hfill \\ {}\hfill & = \mathrm{Prob}\left({V}_{i1}+{\varepsilon}_{i1}>{V}_{i2}+{\varepsilon}_{i2}\right)\hfill \\ {}\hfill & = \mathrm{Prob}\left({V}_{i1}-{V}_{i2}>{\varepsilon}_{i2}-{\varepsilon}_{i1}\right)\hfill \end{array} $$

Given the deterministic parts of the utility functions *V*_*ij*_, this probability depends on the assumptions on the distribution of the stochastic error terms *ε*_*ij*_.

### Choice design

Although a DCE is a quantitative method to model preferences, a qualitative method could be useful to define the attributes and levels of choices when designing the choice set [29]. In order to properly understand CHOs’ motivational preferences, both literature review and pretesting were used to identify sets of motivational factors that are relatively more likely to increase acceptance of community job posting and retention. From several previous studies on job preferences in rural settings, salary, better working conditions, effective support systems, career promotion opportunity, financial incentives, better living conditions, and family support systems have been recognized as major determinants [3, 30–38].

The instrument in this study has eight choice sets with two alternatives. Each alternative has seven attributes: salary, children’s education, equipment, management style, study opportunity, housing, and transportation (Table 2). While some attributes/motivational factors are described quantitatively, others are presented in qualitative terms. For instance, salary is described as “basic salary” and “basic salary plus 50 % of the base salary.” On the other hand, management style is described as “supportive workplace” and “unsupportive workplace.”

**Table 2:** Attributes and levels

Attribute	Level	Description
Salary	1	Base salary plus 50 %
	0	Base salary
Children’s education	1	Allowance for children’s education
	0	No allowance for children’s education
Infrastructure, equipment, supplies	1	Advanced (e.g., reliable electricity, ultrasound, constant drug supply)
	0	Basic (e.g., unreliable electricity, X-ray, intermittent drug supply)
Management style	1	Supportive workplace and management
	0	Unsupportive workplace and management
Years of work before study leave	1	Study leave after 2 years of service
	0	Study leave after 5 years of service
Housing	1	Free housing provided
	0	Housing not provided
Transportation	1	Utility car provided
	0	Utility car not provided

For practical reasons, a fractional-factorial design, which has fewer runs than a full-factorial design, is used to develop an experimental deign in most cases [38]. This study employed an orthogonal fractional-factorial design to ensure efficiency in the design of choice sets. Orthogonal arrays are perfectly efficient because of their both balanced (each attribute level appears equally often) and orthogonal (every pair of levels appears equally often) nature [38]. In this way, eight choice sets are constructed as an orthogonal array, based on the design in Sloane [39].

Table 3 illustrates choice sets as showing the level of attributes assigned to job B for each choice set. The profile of paired job A is a foldover of that of job B, i.e., the mirror image of the design (0 = 1 and 1 = 0). Table 3 also shows the percentage of CHOs who chose job B over job A. In choice set 1, seven CHOs (3.5 %) chose job A over job B, though job B was assumed to be superior to job A. Lancsar and Louviere [40] argued that preferences that may appear to be “irrational” may in reality be compatible with some form of rationality. Since deleting such responses may be inappropriate, all respondents are included in this analysis.

**Table 3:** Attribute levels assigned for choice sets of job B and rate of choice

	Salary	Child education	Equipment	Management	Study leave	Housing	Car	% of CHOs who chose job B over job A
Choice set 1	1	1	1	1	1	1	1	96.5
Choice set 2	0	1	0	1	0	1	0	25.0
Choice set 3	1	0	0	1	1	0	0	68.5
Choice set 4	0	0	1	1	0	0	1	16.0
Choice set 5	1	1	1	0	0	0	0	17.0
Choice set 6	0	1	0	0	1	0	1	55.0
Choice set 7	1	0	0	0	0	1	1	16.5
Choice set 8	0	0	1	0	1	1	0	68.5

### Estimation procedure

The multinomial logit (MNL) might have been the most commonly used model in a DCE [22, 26, 41]. However, the MNL models require three assumptions: independence of irrelevant alternatives (IIA), independent and identically distributed (IID) error terms across observations, and no taste heterogeneity. Because of these assumptions, the MNL is usually criticized as not being an exact representation of choice making [28, 42].

Recent literature shows a clear shift toward more flexible econometric models such as mixed methods [22, 43]. Mixed logit relaxes the assumption of taste homogeneity. According to some systematic reviews of the literature, many studies have found evidence of preference heterogeneity and reported an improved goodness of fit using mixed logit [22, 43]. However, the mixed logit also requires assumptions about the parameters to randomize and the distributions of parameters [29].

Specification tests were performed to determine whether a model allowing a random parameter was appropriate. The selection of random parameters is usually based on either the Lagrange multiplier (LM) test proposed by McFadden and Train (2000) or the t-statistic for the deviation of the random parameter [41, 44, 45]. Either a Wald or likelihood ratio test statistic can be used for the LM test. The t-statistic for standard deviation is commonly used in the literature to determine the random parameters, considering its straightforward and simple application [44].

The LM test by a Wald statistics showed that the chi-square was 0.13, which suggested that we could not reject the hypothesis of no random coefficients. The t-statistic for the standard deviation was also checked with 50 Halton draws (Table 4). The small *p* value in the likelihood ratio test for the joint significance of the standard deviations implies that the null hypothesis that all the standard deviations are equal to zero is rejected [46]. The result in Table 4 shows significant preference heterogeneity for salary, children’s education, equipment, and study leave. Thus, while the LM test does not reject the assumption of no random coefficients, the *t* test results based on the mixed logit model show that these four attributes have preference heterogeneity.

**Table 4:** Result of the specification test (t-stat for standard deviation)

	Coef.	SE	*z*-score	*P* > *z*	95 % confidence interval	
Mean						
Salary	0.52	0.09	5.62	0.00	0.34	0.70
Children’s education	0.46	0.10	4.64	0.00	0.27	0.66
Equipment	0.50	0.09	5.37	0.00	0.32	0.69
Management	0.66	0.09	7.18	0.00	0.48	0.84
Study leave	2.02	0.19	10.88	0.00	1.65	2.38
Housing	0.68	0.09	7.20	0.00	0.50	0.87
Transportation	0.28	0.09	3.31	0.00	0.12	0.45
SD						
Salary	0.34	0.18	1.82	0.07	−0.03	0.70
Children’s education	0.63	0.15	4.34	0.00	0.35	0.92
Equipment	0.42	0.17	2.44	0.02	0.08	0.77
Management	−0.20	0.24	−0.83	0.41	−0.67	0.27
Study leave	1.44	0.17	8.50	0.00	1.11	1.78
Housing	0.15	0.23	0.65	0.52	−0.29	0.59
Transportation	0.08	0.15	0.54	0.59	−0.21	0.37
Log-likelihood	−748.33					
Prob > chi2	0.00					

Although the main-effect designs remain dominant, there has been an increase in the proportion of analyses catering for interactions [43]. De Bekker et al. [22] suggest that future work should explore the inclusion of interaction terms in DCE analyses. Interaction terms between the attributes of alternatives and the choice-invariant socioeconomic characteristics of health workers have been introduced in several previous studies [28, 47, 48].

Regarding the outcome, the use of simulation is useful with DCE data [43]. Such simulation results are potentially useful to estimate the response to job openings. The choice probability is calculated through simulation to approximate the integral. The logit formula *L*_*i*_*(η)* is calculated with draws of *η*. This process is repeated for a certain number of draws. The mean of the resulting *L*_*i*_*(η)*s is taken as the approximate choice probability.

$$ S{P}_i=\frac{1}{R}{\displaystyle \sum_{i=1, \dots,\ R}}{L}_i\left({\eta}^r\right) $$

R is the number of draws of *η*, *η*^*r*^ is the *r*th draw, and *SP*_*i*_ is the simulated probability that an individual chooses alternative *i*.

For these reasons, we conducted mixed logit with and without interaction, using STATA’s mixlogit command, and performed probability simulation. The interaction terms are (a) number of children under 5 in the household, (b) gender dummy (male = 1, female = 0), and (c) years worked at this CHPS as a dummy variable (less than one year = 0, between 1 year and 2 years = 1, and more than 2 years = 2).

## Results

As previously noted, all the CHOs working in three HDSS sites were contacted. None of the CHOs selected refused to be interviewed, and 200 CHOs were interviewed in total. Some of the demographic characteristics of respondents and the background information are presented in Table 5. The average age was 28.1 (SD 6.0), with ages ranging from 22 to 58 years. Most of them were women (84.5 %). The average household size was 2.5. Fifty-three CHOs were staying at the health facility. With respect to the familiarity of the community before they started to work at the current workplace, more than two thirds of them answered they had not known at all or hardly known about the community before they started to work there.

**Table 5:** Selected descriptive statistics of respondents (*N* = 200)

Variable	Description	Freq.	%
Site	Navrongo	54	27.0
	Kintampo	61	30.5
	Dodowa	85	42.5
Age (*n* = 199)	≤25	70	35.0
	26–30	97	48.5
	≥30	32	16.0
Gender	Male	31	15.5
	Female	169	84.5
Marital status	Married or living together	83	41.5
	Other	117	58.5
Religion	Christian	185	92.5
	Other	15	7.5
Ethnicity	Akan	37	18.5
	Ga, Adangbe	22	11.0
	Ewe	25	12.5
	Kassena	30	15.0
	Fulani	42	21.0
	Other	44	22.0
Household size	>1	103	51.5
	0	152	76.0
Number of children under 5	1	34	17.0
	2 or more	14	7.0
Number of years worked at this CHPS	1–2 years	41	20.5
	More than two years	83	41.5
Commute time (*n* = 183)	Stay at health facility	53	26.5
	Less than 15 min	61	30.5
	15–30 min	37	18.5
	>30 min	32	16.0
Familiarity of the workplace	Did not know at all	115	57.5
	Hardly knew	21	10.5
	Knew somewhat	32	16.0
	Knew well	13	6.5
	Knew very well	19	9.5

Attitude to work was also asked to the CHOs. With respect to overall job satisfaction, 70 % of CHOs answered that they were satisfied (very satisfied or satisfied) with their jobs. However, 18.5 % of them were satisfied with their salary. Satisfaction levels with promotion (23 %) and with basic amenities (27 %) were low, whereas that with the content of health service they were providing (73 %) or training (69 %) were high.

The results of the mixed logit model are shown in Table 6.^3^ As mentioned in the “Methods” section, four models were considered. These assumed heterogeneous preferences for four attributes: salary, children’s education, equipment, and study leave. They were all estimated with 50 Halton draws.

**Table 6:** Results from mixed logit estimation

Mixed logit	Model (1)						Model (2)						Model (3)						Model (4)					
Attributes	Main effects only						# of children <5						Male						Years of work					
	Mean	SD	SE	*p* value	95 % CI		Mean	SD	SE	*p* value	95 % CI		Mean	SD	SE	*p* value	95 % CI		Mean	SD	SE	*p* value	95 % CI	
Salary	0.52	−0.37	0.09	<0.01	0.34	0.7	0.43	−0.36	0.1	<0.01	0.23	0.62	0.49	−0.37	0.1	<0.01	0.3	0.68	0.24	−0.37	0.13	0.068	−0.02	0.49
Children’s education	0.46	−0.63	0.1	<0.01	0.27	0.66	0.3	−0.58	0.11	<0.01	0.09	0.5	0.45	−0.62	0.1	<0.01	0.24	0.65	0.36	−0.63	0.15	0.014	0.07	0.65
Equipment	0.49	−0.47	0.09	<0.01	0.31	0.67	0.45	−0.47	0.1	<0.01	0.26	0.65	0.45	−0.46	0.1	<0.01	0.25	0.64	0.48	−0.5	0.14	<0.01	0.21	0.76
Management	0.66		0.09	<0.01	0.48	0.83	0.62		0.1	<0.01	0.43	0.81	0.64		0.09	<0.01	0.46	0.83	0.68		0.13	<0.01	0.42	0.94
Study leave	2.03	−1.42	0.17	<0.01	1.69	2.36	2.03	−1.4	0.18	<0.01	1.68	2.39	1.93	−1.4	0.18	<0.01	1.58	2.27	2.14	−1.44	0.24	<0.01	1.68	2.61
Housing	0.68		0.09	<0.01	0.5	0.86	0.61		0.1	<0.01	0.42	0.8	0.64		0.1	<0.01	0.46	0.83	0.58		0.13	<0.01	0.33	0.83
Transportation	0.28		0.08	<0.01	0.11	0.44	0.23		0.09	0.012	0.05	0.41	0.29		0.09	<0.01	0.12	0.47	0.25		0.13	0.044	0.01	0.5
Interaction terms																								
Salary							0.35		0.16	0.031	0.03	0.67	0.26		0.29	0.384	−0.32	0.83	0.28		0.1	<0.01	0.09	0.47
Children’s education							0.58		0.18	<0.01	0.24	0.93	0.21		0.33	0.535	−0.45	0.86	0.11		0.11	0.303	−0.1	0.32
Equipment							0.15		0.15	0.302	−0.14	0.44	0.42		0.29	0.153	−0.16	1	0.02		0.1	0.871	−0.18	0.21
Management							0.16		0.14	0.255	−0.11	0.43	0.16		0.29	0.569	−0.4	0.72	−0.01		0.09	0.913	−0.19	0.17
Study leave							0.02		0.22	0.927	−0.42	0.46	0.78		0.43	0.068	−0.06	1.62	−0.08		0.15	0.603	−0.38	0.22
Housing							0.29		0.16	0.064	−0.02	0.6	0.3		0.28	0.289	−0.26	0.86	0.11		0.09	0.243	−0.07	0.3
Transportation							0.18		0.13	0.167	−0.08	0.45	−0.04		0.27	0.869	−0.58	0.49	0.03		0.09	0.729	−0.15	0.21
Log-likelihood	−747.59						−739.6						−744.54						−742.33					
Likelihood ratio *χ*2	103.66						100.48						101.45						104.14					

Note: Model (1) estimates only the main effects while Models (2) to (4) include interaction terms—number of children, male, and years of work, respectively

From the main-effect model (model (1)), all attributes turned out to be strongly significant. Study leave after 2 years was a major predictor of preference (*β* 2.03; 95 % CI 1.69 to 2.36). The main-effect coefficients in models (2)–(4) were similar to those in model (1). The interaction terms with number of children in model (2) were positive and significant for children’s education (*β* 0.58; 95 % CI 0.24 to 0.93), salary (*β* 0.35; 95 % CI 0.03 to 0.67), and housing (*β* 0.29; 95 % CI −0.02 to 0.60). Among the male interaction terms in model (3), study leave after 2 years’ service was slightly significant (*β* 0.78; 95 % CI −0.06 to 1.62). From the work-year interaction terms in model (4), those who worked longer at the same CHPS valued salary more than those who worked only a short period (*β* 0.28; 95 % CI 0.09 to 0.47).

Table 7 shows the results of simulation. It presents the predicted probabilities of uptake for rural posting when the attitude level changed from zero to one. For example, if the salary increased by one half, the probability of choosing the job would increase by 6 %. Study leave allowed after 2 rather than 5 years of service had the greatest effect on the probability of accepting the job, which increased by 29.1 %.

**Table 7:** Estimated probabilities with different job attributes

Attributes	Mean	SD	Min	Max
Salary	0.060	0.022	0.009	0.087
Children’s education	0.055	0.022	0.005	0.079
Equipment	0.058	0.021	0.008	0.084
Management	0.076	0.026	0.015	0.106
Study leave	0.291	0.102	0.037	0.377
Housing	0.078	0.027	0.015	0.109
Transportation	0.032	0.012	0.005	0.047

## Discussion

One of the major findings of this study is the importance of early opportunity for study leave. Results from the mixed logit model showed that a relatively shorter period before study leave would be a major predictor of the preference for a particular rural job. As the simulation result showed, if the time period before study leave decreased from 5 to 2 years, the probability of accepting the job would increase by 29.1 %, given the other attributes were equivalent. In a similar study that solicited medical students’ or student midwives’ motivational preference for acceptance of rural posting, participants were willing to accept relocation to rural areas for a limited time, especially if opportunities for further education were attached to the rural service [38, 49].

The value CHOs placed on years before study leave could be due to the implied benefit that they would gain after they upgrade their educational level. Most CHOs knew their promotion opportunities, as observed in the descriptive statistics presented above. Probably, CHOs thought that further study would increase their chances of getting promoted to a higher rank within the health system.

Male CHOs valued shorter periods before study leave more than their female counterparts did. This tendency is consistent with a previous study that showed, by a DCE with clinical officer finalists in Tanzania, that men valued educational opportunities after 2 instead of 6 years more than women did [50].

The relative importance also depended on the CHOs’ socio-demographic characteristics. The mixed logit models with interaction terms confirmed that CHOs with children under 5 placed a higher value on education allowance for children than on other motivational packages. In most cases, CHOs work in communities where access to good schools for their children is limited. This may increase the cost of providing a good education to their children in nearby towns/cities, resulting in a high premium on education allowance for children.

Incentives that could promote health worker acceptance to community postings did not necessarily depend on high-cost policy options such as a large increase in salaries but on motivational policies that would provide them with the opportunity to upgrade their skills and to rise up the professional ladder. Salary was certainly important for all respondents, but a combination of other incentives such as study opportunities could diminish its importance [7]. CHOs might be aware that they will most likely achieve higher futuristic benefits from the opportunity to upgrade their skills. Fewer years of service before further study is a policy option that the government could offer as compared to more financially intensive ones.

On the other hand, CHOs who had been working at the same CHPS for a long time clearly preferred high salary. This implied that salary increase, i.e., use of financial incentives, was an important factor for retention. According to these results, policymakers could allocate their budget strategically. For example, they could recruit health workers to local communities with career development opportunities (study leave after a short period of service) and reward CHOs who have been working in the community for a certain period with salary increase.

## Conclusions

Shorter period of work before study leave was identified as the most important motivation, especially for male CHOs. When years of work were considered, the role of salary stood out. For CHOs with children, allowance for children’s education, salary plus 50 %, and housing provision were valued. In order to reduce health worker shortage in rural settings, policymakers could provide “needs-specific” motivational packages in the form of career development opportunities to attract health workers to community posting and offer financial incentives in the form of salary increase to retain them. We hope these findings would contribute to the policy-making process of making the CHO’s job in communities in Ghana more attractive.

## Abbreviations

CHO(s): Community health officer(s)
CHPS: Community-based health planning and services
CI: Confidence interval
CoC: Continuum of care
DCE(s): Discrete choice experiment(s)
DHRC: Dodowa Health Research Centre
EMBRACE: Ensure Mothers and Babies Regular Access to Care
ERC: Ethics Review Committee
GHS: Ghana Health Service
HDSS: Health and Demographic Surveillance System
HRC: Health Research Center
HQ: Headquarter
IEC: Institutional Ethics Committee
IIA: Independence of Irrelevant Alternatives
IID: Independent and identically distributed
IRB: Institutional Review Board
JICA: Japan International Cooperation Agency
KHRC: Kintampo Health Research Centre
LM: Lagrange multiplier
MDG(s): Millennium Development Goal(s)
MNCH: Maternal Newborn and Child Health
MNL: Multinomial logit
NHRC: Navrongo Health Research Centre
SD: Standard deviation
SE: Standard error
WHO: World Health Organization

## Footnotes

1 The CHPS Initiative is a national program for reorienting and relocating primary health care from sub-district health centers to convenient community locations. Since its inception in 2003, CHPS has been scaled up to a total of 1863 functional zones as of mid-2012.

2 The EMBRACE initiative, launched in 2010, is a strategy to step up Japan’s concerted efforts to help achieve the health-related MDGs in developing countries, particularly regarding maternal and child health. To extend the benefits of the EMBRACE initiative and evidence-based practice in health policy, the Ghana EMBRACE implementation research project was launched in 2012.

3 The conditional logit model was analyzed in the same fashion, but the results are not presented here as they are not substantively different from those of the mixed logit model.
